# Health-seeking behaviour among pregnant women during the COVID-19 pandemic: A qualitative study

**DOI:** 10.1016/j.heliyon.2021.e07972

**Published:** 2021-09-10

**Authors:** David Onchonga, Huda Alfatafta, Enoch Ngetich, Wilbroda Makunda

**Affiliations:** a Doctoral School of Health Sciences, Faculty of Health Sciences, University of Pécs, Hungary; b School of Public Health, Mount Kenya University-Nairobi, Kenya; c Ministry of Health, Kisumu, Kenya

**Keywords:** COVID-19, Health-seeking behaviour, Maternal health, Three delays model, Pregnant women

## Abstract

**Background:**

The novel coronavirus pandemic has killed millions of people globally while significantly destroying the social, economic, and political wellbeing of people. The global pandemic has negatively impacted pregnant women's access to prenatal care. The current study sought to understand the health-seeking behaviour of women who were pregnant during the onset of the COVID-19 pandemic in Kenya.

**Methods:**

The “Three Delay” model theoretical framework was applied to piece together the pregnant women's health-seeking behaviour during the early stages of the pandemic through focus group discussions. The collected qualitative data was analysed using thematic analysis.

**Results:**

The delays in deciding to seek care, delays in reaching healthcare facilities and delays in receiving quality healthcare services at the healthcare facility were a result of the fear of contracting the virus. These delays were occasioned by participants’ personal experiences and uncertainties about COVID-19 pandemic, compulsory quarantines, national cessation of movements, compulsory lockdowns, loss of income to many households and the influence of traditional birth attendants (TBAs).

**Conclusion:**

The current study found that fear of COVID-19 was a major factor that hindered access to maternal healthcare services. In this regard, there is a need to upscale awareness creation on the significance of seeking maternal health services during the pandemic to reduce the possibility of obliterating the gains made in reducing poor health-seeking behaviours among pregnant women.

## 1 Introduction

Prompt access to maternal and child health services has been acknowledged as an essential preventive measure to maternal morbidity and mortality. The inaccessibility of health services has been studied widely [1, 2, 3]. Sadly, the 2019 Coronavirus disease pandemic (2019 SARS-CoV-2) commonly known as COVID-19 has had a substantial influence on the health services delivery globally, including maternal and child health services [4], and consequently led to relatively low uptake of services in the sub-Saharan Africa region and South Asia [5].

According to the recently published studies, pregnant women are not at a higher risk for COVID-19 disease than the general population [6]. For example, the result of 55 pregnant women infected with COVID-19 and 46 neonates were reported with no explicit indication of vertical transmission [7]. However, there is a correlation between COVID-19 and comorbidities among pregnant women [8]. Since the physiological and mechanical changes in pregnancy accelerate the vulnerability to infections, if the cardiorespiratory system is affected, it may lead to rapid progression to respiratory failure among pregnant women [9]. Studies have equally documented mental health issues related to the COVID-19 pandemic [10, 11]. Studies have shown that complications such as Anaemia, haemorrhage, lower limb oedema, laceration, foetal discharge, foetal obstruction, sepsis, fistula, and puerperal psychosis contribute largely to maternal mortality during pregnancy, delivery, and after childbirth.

Pregnant women die as a result of complications following pregnancy, and studies have indicated that it is due to severe bleeding after childbirth, infections during and after birth, pre-eclampsia, and eclampsia, complications from delivery, unsafe abortions, and complications as a result of prenatal fear of childbirth [12]. Although these factors play a key role, there are real reasons that may trigger the fatalities of pregnant women; for example, illiteracy may facilitate the lack of understanding of the importance of seeking maternal health services [13].

Reducing maternal morbidity and mortality continues to be a priority in many countries in the last two decades, and this has successfully yet progressively realized a 38% decrease in global maternal mortality ratio since the year 2000 [14]. Some of the effective interventions for reducing the burden of maternal morbidity and mortality include; access to health facilities with adequately staffed and trained healthcare workers with sufficient skills to recognize, stabilize, and treat/refer the patient, availability of basic and comprehensive emergency obstetric and neonatal care services (BEmONC and CEmONC) in the health facility, an efficient interfacility transfer/referral mechanism [15], and investment in digital mental healthcare innovations more so during emergencies [16].

While there has been a steady improvement in the uptake of maternal and child health services in Kenya, a recently conducted study reported a decrease in routine antenatal care visits to the health facilities, uptake of routine immunization services, a steady decline in the number of skilled birth attendants and a relatively high numbers of stillbirths during the COVID-19 pandemic [17]. Studies undertaken during the previous pandemics, such as Ebola in West Africa [18], have highlighted the same trends.

Thaddeus and Maine's three delays model [19] explicitly highlight three major delays related to maternal mortalities. They include (1) delays in deciding to seek healthcare services, (2) delays in arriving at the health facility and (3) delays in providing adequate healthcare services in health facilities. The first delay has been attributed to socio-cultural dynamics on who makes decisions in the household, the pregnant women's financial ability, and previous negative birth experience [20]. Delays in arriving at health facilities have been reported to be caused by long distances to health facilities, lack of adequate and affordable public and private transport systems, and inaccessibility of the health facilities due to the rough terrains espcially in resource limited settings [21]. The third delay has been explained in the context of lack of or limited human resources for health, inadequacies in medical supplies, and lack of adequate referral systems [1, 22, 23, 24, 25].

During disease outbreaks and pandemics, health-seeking behaviour might change; due to the uncertainties that come with it. The global coronavirus pandemic has impacted the health-seeking behaviours of different cohorts in the population, and there are reports of low uptake of healthcare services. To the best of our knowledge, there are no comprehensive studies done on pregnant women's health-seeking behaviour during the onset of the COVID-19 pandemic. As such, this study was conducted to describe the health-seeking behaviour of pregnant women at the onset of the 2019 COVID-19 pandemic.

The three delays framework was applied to contextualize the study participant's health-seeking behaviour during the onset of the 2019 COVID-19 pandemic. The study had three specific objectives: namely: (i) to understand if there were any delays in making decisions to seek care; in arriving at the health facility, and in the provision of quality health care (ii) to understand the contributing factors (if any) to the three delays and (iii) to understand any barriers to the uptake of maternal health services during the pandemic.

## 2 Methods

### 2.1 Research design and procedure

Qualitative methods of data collection were employed in this descriptive cross-sectional study to piece together the thoughts, feelings, and experiences of pregnant women regarding their health-seeking behaviour during the onset of the COVID-19 pandemic. This was done through the application of the “Three Delays” theoretical framework which was looking at delays in deciding to seek care, delays in reaching healthcare facilities, and delays in receiving quality care at the healthcare facilities. Data collection was done in July and August 2020.

### 2.2 Study settings, population and data collection

The current study enrolled pregnant women who had attended at least one antenatal care clinic (ANC) in a county referal hospital in Kenya. A total of twenty-six pregnant women participated in the present study. This was done through purposive sampling as the study population's demographics were comparatively homogeneous. The inclusion criteria for study participants was (i) having attended at least one ANC visit, (ii) ability to speak in either Swahili or English language, (iii) had lived in the catchment area for at least six months before the onset of the pandemic and (iv) aged between 18-45 years. The first two focus group discussions (FGDs) had seven participants and the last two FGDs had six participants respectively. In the current study, the saturation principle was reached after undertaking the 4^th^ FGD when no new emerging themes were being realized. On average each of the four FGDs took 45–54 min and FGDs were undertaken in four centralized locations where the study participants would access with ease. Four community health workers were trained and tasked with identifying and recruiting the study participants. Two research assistants with a background in midwifery and who had been trained on the scope of the current study undertook the data collection. The research assistants were further assessed on their familiarity with the “Three Delays” theoretical framework that was being employed in the current study.

### 2.3 Focus group discussion

A Focus group discussion guide written in Swahili and English was used to undertake the FGDs. The interview guide was premised on the theoretical framework of the “Three Delays” model as proposed by Thaddeus and Maine [19]. Pre-testing of the data collection tool was done by undertaking one focus group discussion so as (i) to evaluate language competency and content validity of data collection material, (ii) estimate time length for full interview delivery, (iii) to maximize methodological skills and achieving proficiency standards for qualitative data collection and (iv) to assess the feasibility and fidelity of translation and transcription protocol in preparation of the interview text for qualitative analysis. Modifications were done where deemed necessary. All the FGDs were audio-recorded with consent from the study participants and field notes were also taken during the FGDs.

### 2.4 Thematic analysis

The collected data were transcribed verbatim and translated from Swahili to English. The transcripts were read several times and then analysed in details. This study was more explorative and therefore thematic analysis [26] was the most suitable method for data analysis. Initially, codes and themes were generated manually by highlighting the transcripts in segments and categorizing the codes that were realized from the FGDs. This was followed by re-organizing the data according to the emerging model. The third step was to merge codes with more sub-themes and themes, and through an inductive methodology, the relationship between the generated codes, sub-themes and themes was reached. To avoid omitting any relevant and important data, the datasets were re-read again to ensure that the generated themes were in tandem with the initial datasets.

### 2.5 Ethical considerations

The approval to conduct this study was obtained from the Jaramogi Oginga Odinga Teaching and Referral Hospital Ethical Review Committee (IERC/JOOTRH/209/20). The study participants were informed of the objectives of the study and gave informed consent before participating.

## 3 Results

A total of 26 pregnant women took part in the current study and their marital status, level of education, employment status, geographical location and the number of children is presented in Table 1. In this study, the collected data were categorized based on the adopted “Three Delays” theoretical framework. Three main themes with sub-themes emerged from the FGDs. The first theme was delay related to the fear of the COVID-19 pandemic at an individual and community level, which impacted the pregnant women's decision to go for regular antenatal care visits at the health facilities. The second theme was the delays related to reaching health facilities due to restrictions that were put in place to curb the spread of the COVID-19 pandemic. The third theme was delays related to the experience of pregnant women at healthcare facilities. Each of the themes and sub-themes is explained, including direct quotes from the study participants.

**Table 1:** Socio-demographic characteristics of the study participants.

Code	Age	Marital status	Level of education	Employment status	Geographical location	Medical cover	No. of children
SP1	22	Single	Primary	Employed	Rural	yes	1
SP2	34	Married	Secondary	Not employed	Peri-urban	No	1
SP3	25	Single	Primary	Employed	Peri-urban	No	2
SP4	28	Married	Secondary	Not employed	Rural	No	1
SP5	43	Married	College	Not employed	Urban	No	2
SP6	22	Single	Primary	Not employed	Rural	No	1
SP7	21	Single	Secondary	Employed	Urban	yes	2
SP8	27	Married	College	Not employed	Rural	No	1
SP9	36	Married	Secondary	Not employed	Urban	No	2
SP10	33	Married	College	Not employed	Peri-urban	No	1
SP11	31	Married	Primary	Not employed	Rural	No	3
SP12	30	Married	Secondary	Not employed	Peri-urban	No	1
SP13	28	Single	College	Employed	Urban	No	1
SP14	35	Married	Secondary	Not employed	Peri-urban	No	3
SP15	31	Single	College	Not employed	Rural	No	3
SP16	39	Married	Primary	Not employed	Urban	No	1
SP17	33	Married	College	Employed	Rural	No	3
SP18	21	Single	Secondary	Not employed	Peri-urban	No	1
SP19	24	Married	College	Employed	Urban	yes	2
SP20	34	Married	Primary	Not employed	Peri-urban	No	2
SP21	29	Single	Primary	Not employed	Urban	No	2
SP22	20	Married	College	Not employed	Urban	No	3
SP23	21	Single	Secondary	Not employed	Peri-urban	No	3
SP24	31	Single	College	Not employed	Urban	yes	2
SP25	36	Married	Secondary	Not employed	Urban	No	3
SP26	41	Married	College	Not employed	Urban	No	3

### 3.1 Theme 1: delays related to the fear of the COVID-19 pandemic

Delays related to fear of the COVID-19 pandemic was discussed in all the FGDs in details and three sub-themes emerged. The first being the fear at an individual level, the second sub-theme was on the community perceptions regarding the COVID-19 pandemic and the third sub-theme was delays related to personal experience that led to delays in seeking maternal and child health services.

i. Delays as a result of fear of COVID- 19 pandemic at an individual level

Participants mentioned that at an individual level, they feared the uncertainties that came as a result of the rumours and misconceptions about the COVID-19 pandemic. As the news of the pandemic was being aired, study participants mentioned that they took an individual decision not to attend their routine antenatal care clinics as scheduled by the healthcare providers to avoid being infected by the virus.

*“I feared getting infected. I rather stay at home than get infected with the new virus”. SP14.*

*“I have heard a lot about the virus and I will not want to be a statistic”. SP18.*

ii. Delays as a result of fear of COVID-19 pandemic at the community level

Study participants revealed that there were speculations on the adverse effects of the virus at the community level which barred them from seeking routine antenatal care visits. They mentioned that the pandemic had brought fear and stigma at the community level and those who were seen going to hospital were perceived to have contracted the virus. Study participants noted that regular updates from the mass media about the communal spread of the virus in several parts of the country and globally, with some countries having high fatality rates increased their fear of seeking maternal healthcare services. At the time of data collection, they reported that the actual cause of the disease was not yet known and the containment measures that were put in place were alien to them.

*“Everyone in our community is worried about the virus”. SP3*

*“Those who go to the hospital are victimized”. SP8*

*“If they see me going to the hospital, they will badmouth about me”. SP19*

iii. Delays attributed to personal experience

Study participants who had lost family members, close friends, colleagues, and relatives narrated their encounter with the virus as terrifying and tragic. They mentioned instances of some COVID-19 patients put on a life-supporting machine. Other study participants revealed that the COVID-19 pandemic was a lonely disease that denied them the opportunity of spending time with their family members who were admitted to various hospitals. Due to its nature of spread, the family members were not allowed to visit patients.

*“My cousin got infected at the hospital. I don't want to suffer like her”. SP26*

*“The infected person lives a lonely life during isolation. I don't want to be a victim”. SP10*

*“My son was separated from us, I don't want to have the same experience”. SP6*

### 3.2 Theme 2: delays in reaching the health facility

Study participants mentioned that during the pandemic, reaching the health facility was a challenge both in rural, peri-urban, and urban areas. Three sub-themes emerged from the FGDs namely: (i) financial loss, (ii) fear of contracting the virus on the way to the health facility (iii) influence of traditional birth attendants during the pandemic.

i. The financial loss during the pandemic

It was reported that during the pandemic, there was the mandatory cessation of movement from urban areas to rural areas and the partial lockdown slowed down the economy. This affected the study participants directly as those in small businesses were unable to do their work on a full-time basis. It was also reported that small traders lost their stock during the onset of the pandemic as fears and uncertainties were eminent within the community. Four participants mentioned that they were given unpaid leave as their workplaces scaled-down operations. In summary, they noted that the events that preceded the announcement of the first case of COVID-19 pandemic limited their desire and ability to seek routine ANC visits.

*“I shut my business during the corona time, now I don't have money at all”. SP22*

*“My husband lost his job and we were depending on him. Now we have nothing”. SP17*

*“I can't manage to go to the hospital. They are the epicentre of the virus”, SP11*

ii. Fear of contracting the virus on the way to the health facility

The study participants noted that they feared using public transport because they were not assured of social distancing and personal hygiene. They argued that the public transport system was unreliable; often overcrowded and posed a danger of the spread of COVID-19 pandemic. The majority of the study participants noted that they had to walk for long distances to reach the health facility and that other alternative means of transport available became more expensive during the pandemic.

*“Public transport is overcrowded, it is risky using it during this time”. SP21*

*“They say people are dropping down and dying in public transport”. SP10*

*“I can't afford private means to the hospital. Public transport is risky”. SP1*

iii. Influence of traditional birth attendants (TBAs)

The study participants cited that during the partial lockdown, they were unable to access health facilities as a result of the fear of contracting the virus from the health facility and therefore they opted for the services of traditional birth attendants (TBA). They also narrated that they had to walk for long distances to reach the health facilities and there were fears of getting caught in-between the curfew hours as some of the health facilities were understaffed and the waiting time was relatively long. Five participants revealed that they were motivated by their peers who had been assisted by renown TBAs to give birth. They said that the TBAs were very kind, supportive and compassionate; and therefore, they chose their services that were relatively inexpensive compared with the cost of transportation to the health facility and payments for services in the hospital such as registration fees, and purchase of medicines.

*“At the time of curfew, the traditional birth attendants were very helpful”. SP4*

*“The health facilities are far from my home and therefore I opted for the TBA”. SP24*

*“The traditional healers are kind and well respected in the community”. SP23*

### 3.3 Theme 3: delays related to the experience of pregnant women at healthcare facilities

The delays related to the experience of study participants at the health facilities generated three sub-themes namely: (i) attitude of healthcare providers, (ii) overwhelmed health facilities, (iii) challenges with out-of-pocket spending for healthcare services. These sub-themes are highlighted in detail.

i) The attitude of healthcare providers towards pregnant women

It was declared that the main challenge pregnant women faced was the cold reception by healthcare providers in the health facilities during the onset of the pandemic. They illustrated that the most of the healthcare providers were unfriendly to pregnant women especially those who used public transport to reach the health facility. This was said to be a result of the perceived fear of contamination imposed by overcrowding in public transport. Study participants recognized that most healthcare providers were not examining them properly as they used to do in their previous ANC visits before the pandemic.

*“Sometimes theharassment is too much to bear”. SP12*

*“Healthcare workers are abusive and rude to the patients sometimes”. SP15*

*“My previous experience was not pleasing. I will not be comfortable with the same* healthcare provider*”. SP20*

ii) Overwhelmed health facilities

It was said that at the onset of the pandemic, the healthcare workers spent most of their time undertaking training on the management of COVID-19 pandemic. This created an acute shortage of healthcare workers in facilities and as such, there were long waiting hours in health facilities particularly the public health hospitals. Study participants reported that the healthcare workers were overworked and fatigued during the onset of the pandemic as they were also involved in making other processes and procedures to contain the pandemic.

*“There are not enough healthcare workers. It's frustrating to wait for so long”. SP2*

*“The healthcare workers are always away, sometimes the facilities are closed”. SP5*

*“Last time I went but there was no healthcare worker to attendtopatients”. SP7*

iii) Out-of-pocket spending for healthcare services.

The majority of the study participants noted that although maternal and child health services were supposed to be free, they were still required to pay fees for services such as buying the medicines and for inpatient admissions. There were observations from the study participants that those who were unable to pay for services were turned back and this demoralized them and forced some of them to resort to alternatives such as seeking the services of TBAs. The majority of the study participants did not have a medical cover so, they had to pay for services even in public health facilities. The massive loss of jobs at the onset of the pandemic was also mentioned as a major factor that limited the study participant's ability to pay for the needed services.

*“Paying for services is very expensive. I couldn't afford it”. SP13*

*“I don't have insurance cover, so it's expensive going for services”. SP25*

*“services are not always cheap. You have to buy medicines all the time”. SP16*

## 4 Discussion

The current study aimed at piecing together the health-seeking behaviour of pregnant women during the early stages of the COVID-19 pandemic through focus group discussions in the framework of the “Three Delay” model namely: the delays in deciding to seek healthcare services; delays in reaching healthcare facilities; and delays in receiving quality healthcare services while at the healthcare facility.

Firstly, there was fear of the pandemic at an individual level among pregnant women, which was expedited by rumours and misconceptions about the virus. This fear and avoidance of being infected forced the majority of pregnant women to skip their routine antenatal care clinics contrary to schedule. A similar study done during the Ebola Viral Disease (EVD) pandemic in West Africa reported the same finding [18]. Another study done during the Ebola pandemic reported that due to the fear associated with the pandemic, patients would visit health facilities when their health conditions had worsened [27].

At the community level, the COVID-19 pandemic led to fear among pregnant women and from those that participated in the current study, most had withdrawn from seeking health services. The fear of being victimized as the ‘spreader of the virus” in the community limited the pregnant women from going for the services. A study done during the EVD inferred that there was an 18% decrease in women attending the antenatal clinics and an 11% decrease of women attending the health facilities for childbirth [28] due to the fear of stigmatization at the community level.

Participants explained that their individual experience with family members and friends who had been infected with the COVID-19 pandemic contributed greatly to limiting their desire to visit health facilities. Initially, hospitals were assumed to be the epicentre for the virus and so many women had reservations in seeking maternal health services. Similar studies have indicated that during pandemics like the EVD in West Africa; there was a significant decline of service uptake as a result of an individual experience of pregnant women about family members and close friends who had been infected by the EVD [28, 29].

In the current study, it was reported that during the pandemic, reaching the health facility was a challenge both in rural, peri-urban and urban areas due to financial loss, fear of contracting the virus on the way to the health facility and the influence of traditional birth attendants during the pandemic.

In the current study, study participants mentioned that there was a massive loss of jobs and closure of businesses that greatly contributed to a financial burden in seeking maternal healthcare. Studies have shown that large scale outbreaks of infectious diseases can greatly increase morbidity and mortality over a wider geographical area and have the potential of causing significant financial losses to individuals, families [30] and significant threats to the financial stability of many countries in the world [31]; and this may limit affordability of essential services such as maternal and child healthcare services.

Fear of contracting the virus on the way to the health facility was one of the reasons given by the study participants as a contributing factor to delays in reaching the health facility. One study has indicated that during the initial stages of the pandemic, the implementation of unprecedented measures to curb the spread of the virus brought along with fear not only as a result of the restrictions of movement but the uncertainties about contracting the virus while travelling [32].

In the current study, the number of pregnant women seeking the services of traditional birth attendants (TBAs) increased as fear of being exposed to the COVID-19 pandemic at the health facilities continued to dominate. Studies have shown that during previous pandemics such as EVD in West Africa, expectant women sought the services of TBAs more than from the skilled health workers in health facilities [18]. The TBAs play a crucial role in maternal and child health especially in resource-limited settings, as they are trusted by pregnant women and easily accessible. Integrating them into an improved healthcare system as community health workers or as mother-mentors can be an effective way of reducing maternal and perinatal mortality. In some developing countries, the TBAs have been oriented to play a role in accompanying expectant women to health facilities for maternal health services [33].

Delays in the provision of adequate care at the health facilities were as a result of the attitude of healthcare workers, overwhelmed healthcare workers and challenges regarding out-of-pocket spending for health services. Concerning the attitude of healthcare workers, studies have indicated that the attitude of healthcare workers towards pregnant women has an impact on the subsequent uptake of maternal and child health services [34, 35, 36, 37].

It was reported that healthcare workers were overwhelmed during the pandemic because of numerous training that was being undertaken on the management of the COVID-19 pandemic. This acute shortage led to long queues at the health facilities. Study participants who had experienced this delay reported that they were unlikely to attend the subsequent ANC visits. Similar studies have shown that acute shortage of healthcare workers and long waiting hours for health services has the potential of negatively affecting the uptake of essential maternal and child health services and this would potentially reverse the achievements in key healthcare indicators such as the four recommended ANC visits, hospital deliveries and essential immunization services for the new-borns [38, 39, 40].

Out-of-pocket spending for health services was also mentioned as a limiting factor, as the majority of the study respondents were unable to afford extra costs levied on them such as the cost of buying medicines. Studies have indicated that out-of-pocket spending is unreliable especially in developing countries, where majority of pregnant women are unemployed [39, 41, 42, 43].

## 5 Study limitations

This being a qualitative study, the sample size is relatively small and consisted only of those study participants who had visited the health facility at least once for routine ANC visits. The views of those who did not visit health facilities might be different and therefore the results should be generalized with caution.

## 6 Study conclusion

The current study found that fear of the coronavirus was a major factor that hindered access to maternal healthcare services. Other factors such as personal experience with COVID-19 disease, financial losses during the pandemic, fear of contracting the virus at the health facilities and the attitude of healthcare workers during the pandemic necessitated the delays in seeking maternal and child health services. In this regard, there is a need to upscale awareness creation on the significance of seeking maternal health services during the pandemic to reduce the possibility of obliterating the gains made in reducing poor health-seeking behaviours among pregnant women, particularly in resource-scarce settings.

## Declarations

### Author contribution statement

David Onchonga: Conceived and designed the experiments; Performed the experiments; Analyzed and interpreted the data; Wrote the paper.

Huda Alfatafta: Contributed reagents, materials, analysis tools or data; Wrote the paper.

Enoch Ngetich: Conceived and designed the experiments; Performed the experiments; Analyzed and interpreted the data.

Wilbroda Makunda: Analyzed and interpreted the data; Wrote the paper.

### Funding statement

This research did not receive any specific grant from funding agencies in the public, commercial, or not-for-profit sectors.

### Data availability statement

Data will be made available on request.

### Declaration of interests statement

The authors declare no conflict of interest.

### Additional information

No additional information is available for this paper.
